# Recognizing Pattern and Rule of Mutation Signatures Corresponding to Cancer Types

**DOI:** 10.3389/fcell.2021.712931

**Published:** 2021-08-26

**Authors:** Lei Chen, Xianchao Zhou, Tao Zeng, Xiaoyong Pan, Yu-Hang Zhang, Tao Huang, Zhaoyuan Fang, Yu-Dong Cai

**Affiliations:** ^1^School of Life Sciences, Shanghai University, Shanghai, China; ^2^College of Information Engineering, Shanghai Maritime University, Shanghai, China; ^3^School of Life Sciences and Technology, ShanghaiTech University, Shanghai, China; ^4^Center for Single-Cell Omics, School of Public Health, Shanghai Jiao Tong University School of Medicine, Shanghai, China; ^5^CAS Key Laboratory of Computational Biology, Bio-Med Big Data Center, Shanghai Institute of Nutrition and Health, University of Chinese Academy of Sciences, Chinese Academy of Sciences, Shanghai, China; ^6^Key Laboratory of System Control and Information Processing, Institute of Image Processing and Pattern Recognition, Shanghai Jiao Tong University, Ministry of Education of China, Shanghai, China; ^7^Channing Division of Network Medicine, Brigham and Women’s Hospital, Harvard Medical School, Boston, MA, United States; ^8^Key Laboratory of Tissue Microenvironment and Tumor, Shanghai Institute of Nutrition and Health, Chinese Academy of Sciences, Shanghai, China; ^9^Zhejiang University-University of Edinburgh Institute, Zhejiang University School of Medicine, Haining, China

**Keywords:** cancer, subtype, mutation signature, pattern, rule, classification

## Abstract

Cancer has been generally defined as a cluster of systematic malignant pathogenesis involving abnormal cell growth. Genetic mutations derived from environmental factors and inherited genetics trigger the initiation and progression of cancers. Although several well-known factors affect cancer, mutation features and rules that affect cancers are relatively unknown due to limited related studies. In this study, a computational investigation on mutation profiles of cancer samples in 27 types was given. These profiles were first analyzed by the Monte Carlo Feature Selection (MCFS) method. A feature list was thus obtained. Then, the incremental feature selection (IFS) method adopted such list to extract essential mutation features related to 27 cancer types, find out 207 mutation rules and construct efficient classifiers. The top 37 mutation features corresponding to different cancer types were discussed. All the qualitatively analyzed gene mutation features contribute to the distinction of different types of cancers, and most of such mutation rules are supported by recent literature. Therefore, our computational investigation could identify potential biomarkers and prediction rules for cancers in the mutation signature level.

## Introduction

Cancer is generally defined as a systematic disease with abnormal cell proliferation and invasion potentials. The general symptoms of cancer include cough, weight loss, lump, and abnormal bleeding, depending on the pathological regions of cancer. Such symptoms are common but not specific in cancers, which may also be shared in other diseases. Genetic factors from either noxious environmental factors or inherited variations trigger the initiation and progression of cancers (Anand et al., 2008; Tang et al., 2018). The regulation and functioning of genetic alterations vary at different omics levels with alternative pathological potentials. However, one of the shared and most common pathological alterations at the phenotypic level is abnormal cell proliferation, which is consistently regulated at the transcriptomics and proteomics level, apart from genomic regulations (Feitelson et al., 2015; Li Z. et al., 2020). Generated from and regulated by tumor microenvironment, cancer is consistently shaped by surrounding cells and tissues, making tumor microenvironment a crucial factor for tumorigenesis in most kinds of cancers, including lung, colorectal, cervical, and breast cancers.

Various biological or pathological behavior affect cancer during initiation, progression, and metastasis. For instance, a bidirectional functioning has been identified on autophagy, one of the most common biological behavior during tumorigenesis. Autophagy suppresses malignant transformation during cancer initiation (Kondo et al., 2005); however, with the invasion and progression of cancer, autophagy may promote the malignant proliferation of cancer cells (de Visser et al., 2006; Nadia and Ramana, 2020). Apart from biological processes, such as autophagy, cancer immune responses are another key factor affecting cancer initiation, progression, and metastasis. Complicated relationships between immune cells and general tumor biological processes, such as initiation, progression, and metastasis, have been recognized in multiple cancer subtypes. Similarly, oxygen-mediated cell damage (Srinivas et al., 2019), growth factor abnormality (Normanno et al., 2017; Umino et al., 2018), and various similar malignant alterations have been recognized during tumorigenesis, describing a complicated profiling of tumor biology at different omics levels.

Clustering cancer related SNPs have been one of the most important research fields in cancer biology for a long time. Multiple previous clustering methods have been presented to summarize the mutational patterns of different cancers. According to one of the most famous cancer mutation databases, COSMIC (Catalogue of Somatic Mutations In Cancer) (Tate et al., 2019), three groups of mutational signatures, including Single Base Substitution (SBS), Doublet Base Substitution (DBS), and Small insertions and deletions (ID), have been summarized. However, such clustering only involves in the molecular biological features of cancer mutations without functional interpretation. Furthermore, in 2019, investigators from University Medical Centre Utrecht summarized current contribution of cancer mutations on clinical diagnosis and identified functional interpretations of existed identified cancer mutation biomarkers (Van Hoeck et al., 2019). However, such results only summarized the effects of mutations with validated functional interpretation. As for computational contribution of cancer mutation clustering, few methods have been presented and these algorithms can only recognize single nucleotide variants (SNVs) and copy number variants (CNVs) (Maura et al., 2019), but not mutation patterns involving multiple base pairs. Therefore, as a summary, the method we presented here have two major innovations: (1) we are targeting essential malignant signatures at the level of mutation patterns not just SNVs or just CNVs; (2) we established quantitative rules to evaluate the contribution of mutation patterns to tumorigenesis.

Among such biological characteristics, cancer-associated genetic mutations are one of the key and essential malignant signatures related to the initiation, invasion, and metastasis of cancers. Specific mutation patterns (ACA to AAA, ACC to AAC, ACG to AAG, etc.) on target regions/genes of the genome have been widely reported to participate in tumorigenesis. For instance, mutant *KAI1*, regulating a batch of downstream tumor-associated genes, has been observed during metastasis in human prostate cancer (Dong et al., 1996; Yang L. et al., 2020). hMLH1 functioning in DNA mismatch repair is one of the most important molecular biomarkers for hereditary non-polyposis colon cancer at the genomics level (Bronner et al., 1994; Ran et al., 2020). The frequency of *Smad4* gene mutation in human colorectal cancer is higher than in any type of cancer (Miyaki et al., 1999; Zhang et al., 2020). Other genes, such as P53 (Fujimoto et al., 1992) and VHL (Kim and Kaelin, 2004), influence cancer development to some extent. However, studies on the mutation features and rules that affect cancers are limited. In the present study, we gave a computational investigation on mutation profiles of cancer samples in 27 types. The powerful feature selection method, Monte Carlo Feature Selection (MCFS) (Dramiński et al., 2007), was adopted to analyze such profiles. A feature list was generated. Then, the incremental feature selection (IFS) (Liu and Setiono, 1998) was applied to this list to extract essential mutation features, find out interesting rules and construct efficient classifiers. As a result, 207 rules and many mutation features related to the 27 types of cancers were obtained. We discussed the top 37 mutation features corresponding to different types of cancers. Our study may serve as a reference in establishing a novel qualitative and quantitative standard in identifying tumor type-specific mutation patterns for tumor classification, and thus provide a new tool for the study of tumorigenesis mechanism based on mutation signatures.

## Materials and Methods

### Datasets

We downloaded the relative mutation frequency of 96 mutation types in 2,892 patients from 27 cancer types (Lawrence et al., 2013). The sample sizes of each cancer type are listed in Table 1. The relative mutation frequency of each mutation type in each cancer patient was calculated by Lawrence et al. (2013) and defined as $R_{cs}=\left(\frac{n_{cs}}{N_{cs}}\right)/\left(\sum_{c}n_{cs}/\sum_{c}N_{cs}\right)$, where *s* is the sample, *c* is the mutation type, *n*_*cs*_ is the number of observed mutations, and *N*_*cs*_ is the number of bases with enough coverage (≥14 reads in tumor cases and ≥8 reads in normal cases) to observe mutation. The mutation types were summarized according to their base pair changes identified in a data set of 3,083 tumor–normal pairs across 27 tumor types (Lawrence et al., 2013). Mutations were specified in the middle of three base pair patterns with all possible mutational directions. The detailed mutation types are provided in Supplementary Table 1. Several studies have suggested that the mutation signatures of different cancers vary and involve combinations of the above mutation types (Alexandrov et al., 2013; Lawrence et al., 2013; Huang et al., 2018; Wojtowicz et al., 2019). We investigated the cancer mutation signatures quantitatively through advanced machine learning methods and identified the mutation rules for explaining and understanding each cancer type.

**TABLE 1:** Sample sizes of 27 cancer types.

Index	Cancer type	Sample size
1	Acute myeloid leukemia	119
2	Bladder	35
3	Breast	120
4	Carcinoid	21
5	Cervical	20
6	Chronic lymphocytic leukemia	87
7	Colorectal	230
8	Diffuse large B-cell lymphoma	49
9	Esophageal adenocarcinoma	76
10	Ewing sarcoma	9
11	Glioblastoma multiforme	213
12	Head and neck	165
13	Kidney clear cell	212
14	Kidney papillary cell	11
15	Low-grade glioma	55
16	Lung adenocarcinoma	327
17	Lung squamous cell carcinoma	177
18	Medulloblastoma	16
19	Melanoma	121
20	Multiple myeloma	62
21	Neuroblastoma	61
22	Ovarian	382
23	Pancreas	9
24	Prostate	196
25	Rhabdoid tumor	3
26	Stomach	87
27	Thyroid	29

### Feature Selection

We applied feature selection to discriminate influential mutation types from the unrelated ones in the dataset. First, MCFS (Dramiński et al., 2007) was used to evaluate the importance of each mutation type. A feature list was generated. Then, a set of optimal mutation types with strong distinctions between different cancer types was obtained by applying IFS (Liu and Setiono, 1998; Ma et al., 2020) with a supervised classifier on such list.

#### Monte Carlo Feature Selection

In this study, we used the MCFS method to assess the importance of mutation types. MCFS is a feature selection method based on the random features of the original features (Dramiński et al., 2007). Given a dataset with *d* features (in this study, 96 mutation types were deemed as features), MCFS first randomly constructed *p* feature subsets, each of which contains *m* features, where *m* is much smaller than *d*. Second, for each feature subset, *t* decision trees are built. Each tree is constructed based on 66% samples that are randomly selected from the original dataset, and the rest samples are used to test such tree. Thus, *p* × *t* trees can be constructed in total. Based on these trees, the importance of each feature, called relative importance (RI) score in MCFS, is evaluated by the following equation

(1) $$RI_{f}=\sum_{\tau =1}^{pt}{\left(wAcc\right)}^{u}IG\left(n_{f}\left(\tau \right)\right){\left(\frac{no.in\;n_{f}\left(\tau \right)}{no.in\tau }\right)}^{v},$$

where wAcc is the weighted accuracy of the decision tree τ and *n*_*f*_(τ) is a node of feature *f* in decision tree τ. The information gain of *n*_*f*_(τ) is expressed as *IG*(*n*_*f*_(τ)), and *no*.*in n*_*f*_(τ) is the number of training samples in *n*_*f*_(τ). *u* and *v* are two different weighting factors.

The MCFS program used in this study was retrieved from http://www.ipipan.eu/staff/m.draminski/mcfs.html. For convenience, default parameters were adopted. In detail, *u* = *v* = 1, *p* = 3,000, *t* = 5, *m* = 5. The 96 features (mutation types) were analyzed by the MCFS program. Each feature was assigned a RI score. Evidently, a feature with a high RI score was more important than that with a low RI score. Thus, we sorted all 96 features with the decreasing order of their RI scores. For formulation, this list was denoted by *F*.

#### Incremental Feature Selection

Incremental Feature Selection (IFS) (Liu and Setiono, 1998) is a feature selection method that filters out a set of optimal features to accurately distinguish different sample classes. As mentioned in section “Monte Carlo Feature Selection,” a feature list *F* was generated by MCFS method. Clearly, the high-ranked features should have positive contributions to classification and can help the classification algorithm to produce good performance. To perform IFS, we first created a series of feature subsets with a step 1 from the feature list *F*. In detail, the first feature subset included the top feature in the list *F*, the second feature subset contained the top two features, and so forth. For each constructed feature subset, a random forest (RF) classifier was built based on samples represented by features in the subset and it was further evaluated by 10-fold cross-validation (Kohavi, 1995; Li J. et al., 2020; Zhou et al., 2020; Liu et al., 2021; Pan et al., 2021; Zhang et al., 2021a, b, c; Zhu et al., 2021). After testing all feature subsets, we obtained the optimal feature subset with the optimal performance. This feature subset was termed as the optimal feature subset and the classifier with such subset was called the optimal classifier.

### Synthetic Minority Over-Sampling Technique

Synthetic Minority Over-Sampling Technique (SMOTE) (Chawla et al., 2002; Chao et al., 2019; Yang X.F. et al., 2020) is a classic technology used to address the potential sample imbalance issue during classification learning. SMOTE can add new samples into the minority class as the same number of samples in the majority class, in an oversampling manner. SMOTE includes several computational steps: (1) it randomly selects a sample *x* in the minority class; (2) it finds *k* neighboring samples in such class with *x*; (3) it randomly select again a sample *y* from these neighboring samples to generate a new sample *z* by a linear combination of *x* and *y*; (4) it places each new sample *z* into the minority class; and (5) it repeats the above steps with predefined times. We directly adopted SMOTE in WEKA (Witten and Frank, 2005).

When evaluating the performance of classifiers in the IFS method, SMOTE was used to decrease the influence of imbalanced problem. In detail, in each round of 10-fold cross-validation, the training dataset was processed by SMOTE so that all classes had same number of samples. The classifier was built on such dataset and further used to predict testing samples.

### Random Forest

A RF is a classifier that is a predictive model for establishing classification and regression problems. It determines the output class for one sample by aggregating votes from different decision trees (Breiman, 2001). As one of the common methods of machine learning, we built a RF by constructing a large number of decision trees. Averaging the predictions of all decision trees to reduce the variance will slightly increase the bias of the predictions, but at the same time, the performance of the model will be considerably improved while avoiding over-learning. As one of the common methods in the field of machine learning, it has wide applications in tackling different biological problems (Pugalenthi et al., 2011; Pan et al., 2014; Marques et al., 2016; Zhao X. et al., 2018; Ru et al., 2019; Zhang et al., 2019; Zhao R. et al., 2019; Zhao X. et al., 2019; Jia et al., 2020; Liang et al., 2020). In this study, we used the tool “RandomForest” in Weka (Witten and Frank, 2005), which implements the RF. Default parameters were used, where the number of decision tree was 10.

### Rule Learning

In this study, we also used the interpretable machine learning method repeated incremental pruning to produce error reduction (RIPPER) (Cohen, 1995) to learn the classification rules. In RIPPER, each rule is an IF-ELSE statement; for instance, if gene1 > 1.3 and gene2 < 5, then breast cancer occurs. The learned rules can be used to make predictions for new samples. We used the RIPPER implemented tool “JRip” in WEKA.

### Performance Measurement

Matthew’s Correlation Coefficient (MCC) (Matthews, 1975) is a commonly used method for estimating performance measurements of classification models (Chen et al., 2017a, b, 2018; Cui and Chen, 2019). However, the original MCC was designed for binary classification problem. In this study, 27 cancer types were involved. Thus, we used the MCC in multi-class version, which was proposed by Gorodkin (2004). To calculate such MCC, two matrices *X* and *Y* must be constructed first, where *X* stands for the true labels of all samples and *Y* represents the predicted labels of all samples. Then, the MCC in multi-class can be computed by

(2) $$MCC=\frac{cov\left(X,Y\right)}{\sqrt{cov\left(X,X\right)cov\left(Y,Y\right)}},$$

where *cov*(⋅) indicates the correlation coefficient of two matrices. Similar to the original MCC, MCC in multi-class ranges between –1 and 1. A high MCC implies the good performance. For convenience, we still called MCC in multi-class as MCC in the following text.

Besides, we also reported the accuracy of each cancer type and overall accuracy to give a complete picture on the performance of each classifier.

## Results

**FIGURE 1:**
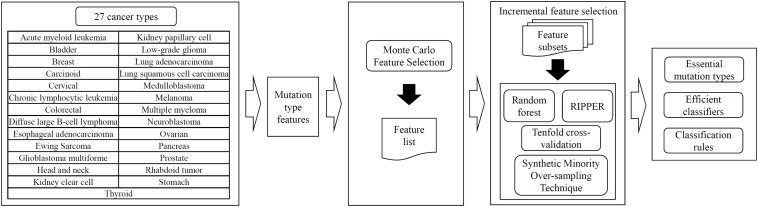
Entire procedures for the investigation on mutation profiles of cancer samples in 27 types. The profiles are analyzed by the MCFS method, resulting in a feature list. Such list is fed into the incremental feature selection, incorporating random forest or RIPPER as the classification algorithm, to extract essential mutation types, build efficient classifiers and construct classification rules.

In this study, several machine learning methods were adopted to investigate the mutation profiles of cancer samples in 27 types. The entire procedures are illustrated in Figure 1.

### Results of MCFS Method

The mutation profiles were first analyzed by the MCFS method to assess the importance of each mutation type. Each mutation type was assigned a RI score, which is listed in Supplementary Table 2. Then, all mutation types were sorted by the decreasing order of their RI scores, resulting in a feature list *F*. Such list is also provided in Supplementary Table 2.

### Results of IFS Method

**FIGURE 2:**
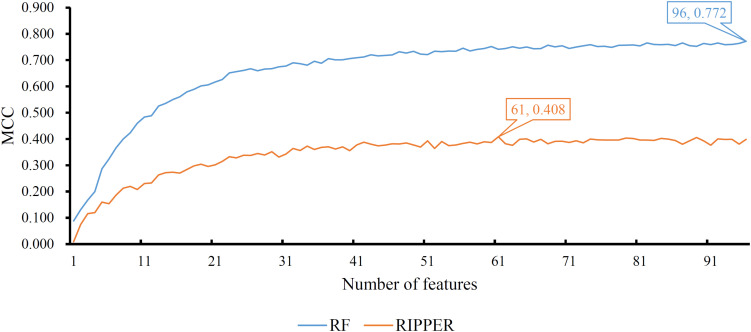
Performance of RF and RIPPER on different feature subsets. The RF yields the highest MCC of 0.772 when all features are used, whereas RIPPER generates the highest MCC of 0.408 when top 61 features are used.

**FIGURE 3:**
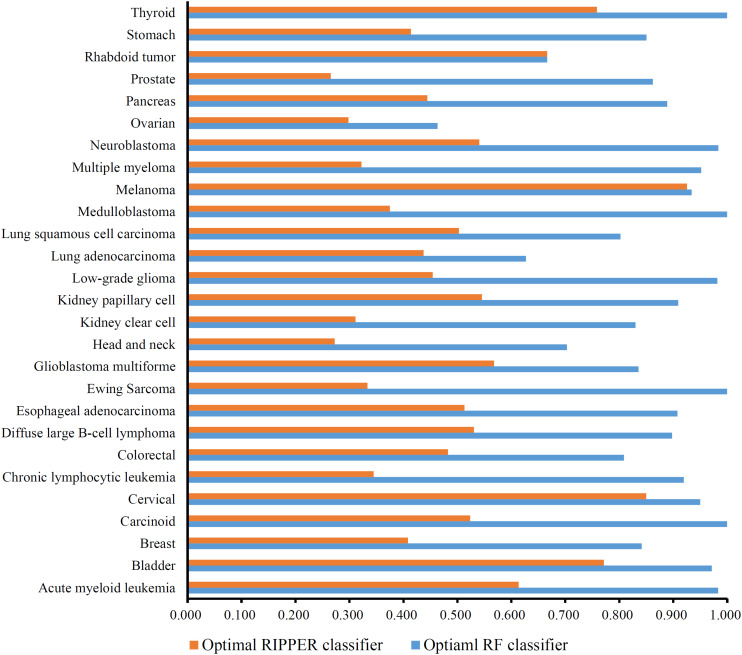
Accuracy on each cancer type yielded by the optimal RF and RIPPER classifiers. The optimal RF classifier gives high performance (accuracy > 0.900) on 14 cancer types, indicating its good performance. The optimal RIPPER classifier provides much lower performance.

A feature list *F* was generated according to the results of MCFS. Such list was fed into the IFS method. Two classification algorithms: RF and RIPPER, were integrated in the IFS method. For RF, its performance on each feature subset is provided in Supplementary Table 3. An IFS curve was plotted, as shown in Figure 2, for an easy observation. The number of features was set as X-axis and MCC was set as the Y-axis. It can be observed that the highest MCC was 0.772 when top 96 features were adopted. Surprisingly, all mutation features were used in this case, indicating that each mutation type gave less or more contributions to the distinction of different cancer types. Accordingly, all mutation features comprised the optimal feature subset for RF and the RF classifier with these features was called the optimal RF classifier. The overall accuracy of such classifier was 0.784, as listed in Table 2. Its detailed performance on 27 cancer types is illustrated in Figure 3. 14 cancer types received the accuracies higher than 0.900. All these suggested the good performance of such RF classifier. Furthermore, we also employed the rule learning algorithm, RIPPER, to do the same procedures. The performance of RIPPER on all feature subsets is also available in Supplementary Table 3. Likewise, an IFS curve was plotted, as illustrated in Figure 2. Evidently, the highest MCC was 0.408 when top 61 features were adopted. Subsequently, the optimal RIPPER classifier was constructed using these 61 features and these features constituted the optimal feature subset for RIPPER. The MCC (0.408) was much lower than that of the optimal RF classifier (0.772). The overall accuracy was 0.443, as listed in Table 2, also much lower than that of the optimal RF classifier (0.784). The detailed performance of the optimal RIPPER classifier on all cancer types is illustrated in Figure 3. It can be observed that almost all cancer types received lower accuracies than those of the optimal RF classifier. Thus, the optimal RF classifier was much superior to the optimal RIPPER classifier. However, the optimal RF classifier was an absolute black-box classifier, few insights can be extracted from such classifier. The RIPPER classifier was much better in this regard because it can learn some classification rules for explaining and understanding particular cancer differences. In detail, generally, several mutation features were involved in one classification rule that can be used to predict one cancer type. These mutation features together with their corresponding thresholds can comprise a mutation pattern on such cancer type. Further investigation on such pattern was helpful to understand the mechanism of this cancer type in the mutation signature level.

**TABLE 2:** Performance of IFS with RF and RIPPER for classifying samples from different cancers.

Classifier	Number of features	Overall accuracy	MCC
RF	96	0.784	0.772
RIPPER	61	0.443	0.408

**FIGURE 4:**
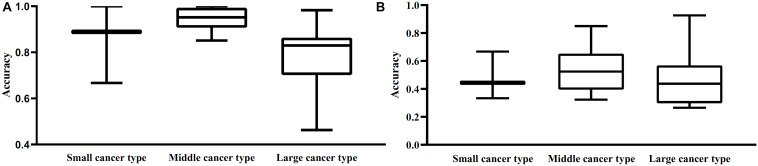
Box plot to show the performance of the optimal RF and RIPPER classifier on three different cancer types. Small cancer type includes the types with less than 10 samples; middle cancer type contains types containing 10–100 samples; larger cancer type contains types with more than 100 samples. **(A)** Box plot for the optimal RF classifier; **(B)** Box plot for the optimal RIPPER classifier. The performance on middle cancer type of two optimal classifiers is best.

From Table 1, we can see that some cancer types contained much more samples than other types, that is, the type sizes were of great differences. Here, we investigated the performance of two optimal classifiers on cancer types with different sizes. To this end, we classified 27 cancer types into three categories. The first category contained types with less than 10 samples, the second category included the types with 10–100 samples, and the third category contained types with more than 100 samples. For convenience, these three categories were called small, middle and large cancer types. The performance of the optimal RF and RIPPER classifiers on three categories is illustrated in Figure 4. It can be observed that the middle cancer type received relative higher accuracies, whereas the small cancer type received slightly higher accuracies than the large cancer type. The reason may be the application of SMOTE. It can increase the performance on minor classes (small cancer type) and decrease the performance on major classes (large cancer type).

### Classification Rules

**FIGURE 5:**
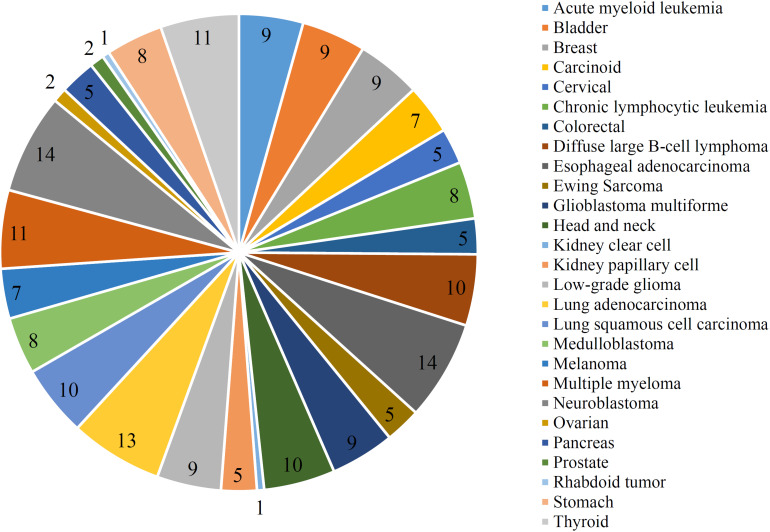
Pie chart to show the number of rules on each cancer type.

The optimal RIPPER classifier used the top 61 mutation features. Accordingly, the RIPPER was applied on all cancer samples that were represented by these 61 features. As a result, 207 rules were obtained, which are provided in Supplementary Table 4. Each cancer type had at least one rules. The number of rules on each cancer type is shown in Figure 5. The cancer types “Esophageal adenocarcinoma” and “Neuroblastoma” had most rules, whereas “Kidney clear cell” and “Rhabdoid tumor” had only one rule. In section “Mutation Pattern Rules Associated With Cancer Subgrouping,” some representative rules would be discussed.

## Discussion

We summarized optimal features describing the distinct mutation types to generate an applicable classifier for all the 27 cancer subtypes. The contribution of typical mutation patterns was functionally connected to certain cancer biological behavior, such as the initiation and progression of tumorigenesis. The top 37 features were supported by previous studies as biomarker candidates. In addition to these potential cancer biomarkers, we identified 27 rules of mutation types associated with these 27 cancers. Such group of quantitative rules may contribute to the detailed classification of cancers and serve as guidance in future research.

### Mutation Patterns Associated With Cancer Subgrouping

First, we screened a group of functional mutation patterns with different distributions in various cancer types. Recent publications confirmed such mutation patterns consistent with our prediction. Here, we selected the top five mutation patterns for following detailed discussions.

The top mutation pattern ACG to ATG refers to amino acid substitution from Thr to Met. Recent publications indicated that such mutation pattern exists in multiple cancer-associated genes, including TP53, PRSS1, and XRCC3. For TP53, such mutation pattern has been recognized in multiple tumor types, especially for head and neck cancer (Boyle et al., 1993) and lung cancer (Veldore et al., 2015), rather than other malignant proliferative diseases, such as esophageal adenocarcinoma and melanoma; this finding reflects the distinctive potentials of such mutation pattern. For PRSS1, such mutation pattern has only been found in pancreatic cancer, validating our prediction (Yi et al., 2016). Similarly, XRCC3 has such a mutation pattern in multiple cancer types, including thyroid cancer (Wang et al., 2015) and breast cancer (Lee et al., 2007). Therefore, as we have mentioned above, this mutation pattern has been discovered in limited tumor types but not in all tumor categories, validating our prediction of mutation pattern distinctive with certain tumor types.

CCG to CTG, indicating the transition from Pro to Leu, is another mutation pattern we identified. According to previous studies, such mutation pattern in protein IL-10 contributes to the anti-tumor immune responses in prostate cancer but not in other tumor types, corresponding with our prediction (Bandil et al., 2017). Such mutation pattern has also been identified in the gene p16 in multiple tumor types but not in all of our candidate tumor types (Zhang and Peng, 2002), further validating our analysis.

The mutation pattern TCG to TTG describes the amino acid transition from serine to leucine. According to recent reports, such mutation pattern may also distinguish certain tumor types from the other ones, which has been validated to be pathogenic in a specific oncogene named RET for thyroid carcinoma (Colombo-Benkmann et al., 2008), confirming our prediction. Further, SMAD2 and SMAD4 as the two essential components of the TGF beta-Smad signaling pathway also have such variant pattern contributing to the tumorigenesis of head and neck squamous cell carcinoma (Qiu et al., 2007) in contrast with other tumor types.

Apart from the optimal mutation patterns analyzed above, the mutation patterns from GCG to GTG (i.e., from alanine to valine) and from TCA to TTA (i.e., from serine to leucine) (Zhang and Peng, 2002; Bandil et al., 2017) have been distinctively identified in different tumor types, which is consistent with to our prediction.

### Mutation Pattern Rules Associated With Cancer Subgrouping

A total of 207 mutation rules are related to 27 cancers. This work focused on a few representative cancer types and several top mutation rules for each cancer.

#### Melanoma

The first rule is the mutation of codon CCC to CTC (Leu to Pro). In 1998, it was reported that the case of melanoma is particularly peculiar, showing Leu-to-Pro mutations in codons 31 and 35, both of which are located in the highly conserved regions (Kumar et al., 1998). P53 mutations in melanoma cell lines, metastases, and primary tumors include the Leu-to-Pro mutations (Zerp et al., 1999). The second rule is the mutations of codons from CCC to CTC, TCC to TTC, TAA to TTA, and the third rule includes the mutations from CCC to CTC, and CCG to CTG, which are some refinements of the first rule.

#### Head and Neck

With typical symptoms as a lump or sore, head and neck cancer is a general description of all cancers related to mouth, nose, and other accessory organs around the head. The first rule for head and neck cancer involves mutations of codons TCA to TTA (Ser to Leu), CAG to CGG (Glu to Arg), and TAT to TGT (Cys to Tyr). In 2007, researchers identified an effective variation on SMAD2. Such variation located in exon 8 at codon 276 (Ser to Leu) contributes to the progression of human head and neck squamous cell carcinoma. The mutations of SMAD2 disrupts a famous tumor associated pathway named transforming growth factor β-Smad signaling pathway (Qiu et al., 2007). The second rule in head and neck cancer involves the mutation of codons TCA to TGA (Ser to a stop codon), ACG to ATG (Thr to Met), AAG to AGG (Lys to Arg), GAG to GGG (Glu to Gly), and TCC to TTC (Ser to Phe). TP53 mutation rate increases following the development and progression of head and neck cancer, and Thr to Met is a well-known p53 mutation (Boyle et al., 1993). The third rule in head and neck cancer includes mutations from codons CCG to CAG, GAC to GGC, and GCT to GTT; these mutations are especially related to CYP1 gene, and the genetic polymorphisms of CYP are associated with head and neck cancer (Gattás et al., 2010).

#### Esophageal Adenocarcinoma

Arising from the esophagus, esophageal adenocarcinoma is one of the most common subtypes of malignancies affecting the digestive tract. The clinical symptoms of such cancer include difficulty in swallowing and weight loss. The first rule in esophageal cancer involves six mutations, such as AAG to ACG, CAT to CGT, GCC to GGC, CCG to CAG, GCA to GAA, and ACA to ATA. A highly significant association exists between P53 mutations in the molecular pathogenesis of esophageal adenocarcinoma and esophageal malignancy, and AAG to ACG is a remarkable type of TP53 mutation (Vaninetti et al., 2008). The second rule in esophageal adenocarcinoma comprises mutations, such as TAA to TTA, TCT to TGT, GCT to GTT, and CCT to CAT. The third rule involves mutations, such as AAG to AGG, CAT to CGT, CAG to CTG, TCG to TAG, and ACC to AAC. The missense polymorphism of human AGT gene (at codon 276, Ser to Leu) is pathogenic for such disease, revealing the specific characteristics of such diseases at the mutational pattern level. In addition, we confirmed the existence of a codon 84 genetic polymorphism previously, which converts leucine to phenylalanine (Deng et al., 1999).

#### Neuroblastoma

With cancer-associated bone pain, neck, and chest lump as typical clinical symptoms, neuroblastoma derives from nerve tissues with high cellular diversity. The first rule in neuroblastoma is the involvement of five mutations, including ACG to ATG, GCA to GAA, CAC to CCC, CCA to CTA, and TCC to TTC. The second rule comprises the mutations, such as CAC to CCC, TCC to TAC, ACA to ATA, and CCA to CAA. The third comprises the mutations ACA to AAA, TCA to TTA, TCT to TAT, GCC to GTC, and ACC to ATC.

### Comparison With COSMIC Database

Here, we further compared our results with previously reported effective mutation patterns in COSMIC database with solid publication supports. Although in COSMIC, the mutations are summarized based on single base not a combination of three constitutive bases, we did find the consistency of COSMIC validated mutation patterns and our results.

For instance, in melanoma, we identified CCC to CTC, TCC to TTC, and CCG to CTG as three typical mutation patterns, which all involved specific cosmic mutation type as C > T. In COSMIC database, more than 23% of patients have such mutation pattern in melanoma associated genomic regions (Tate et al., 2019). Apart from that, such mutation patterns have been identified in melanoma associated genes, like OR4F5 and SAMD11, validating the specific role of such mutational patterns in melanoma.

Furthermore, as for esophageal adenocarcinoma, mutation patterns like GCA to GAA, CCT to CAT, and TCG to TAG are significant mutations identified in this study with the same single base pair alterations as C > A. According to COSMIC database, C > A pattern has been shown to be identified in more than 54% of all patients associated with esophageal adenocarcinoma (Tate et al., 2019), indicating the specific biological functions of such mutation patterns on such cancer subtypes.

Apart from mutation patterns associated with specific cancer subtypes, we also identified a group of effective mutation patterns associated with APOBEC (Chen et al., 2019). As an apolipoprotein B mRNA editing enzyme, APOBEC family has been shown to be associated with multiple cancer mutations and contribute to the variety of cancer mutation burdens. In our study, we also identified some specific APOBEC associated mutation patterns like TCG > TTG and TCA > TTA, validating that identified mutation patterns are associated with the initiation and progression of different cancer subtypes.

Therefore, the mutation patterns identified in this study to be associated with different cancer subtypes have been validated by COSMIC database, implying reliability of our results.

## Conclusion

All of the qualitatively analyzed mutation signatures contribute to the distinction of different types of cancers. Most of the quantitative analyzed mutation rules are supported by recent literature. Our computational approach could efficiently identify mutation signatures and rules for cancers.

## Data Availability Statement

Publicly available datasets were analyzed in this study. This data can be found here: https://www.nature.com/articles/nature12213.

## Author Contributions

TH, ZF, and Y-DC designed the study. LC, XZ, and XP performed the experiments. TZ and Y-HZ analyzed the results. LC and XZ wrote the manuscript. All authors contributed to the research and reviewed the manuscript.

## Conflict of Interest

The authors declare that the research was conducted in the absence of any commercial or financial relationships that could be construed as a potential conflict of interest.

## Publisher’s Note

All claims expressed in this article are solely those of the authors and do not necessarily represent those of their affiliated organizations, or those of the publisher, the editors and the reviewers. Any product that may be evaluated in this article, or claim that may be made by its manufacturer, is not guaranteed or endorsed by the publisher.

## Supplementary Material

Supplementary Table 1 Information of 96 mutation types.

Supplementary Table 2 RI scores of mutation types.

Supplementary Table 3 IFS corresponding to different numbers of features used.

Supplementary Table 4 Classification rules generated by RIPPER.
